# Books Reviewed

**Published:** 1862-11

**Authors:** 


### BOOKS REVIEWED.

*Variola; its Nature and Treatment, with an Addendum. By* ANDREW NEBINGER, M. D. *Philadelphia*, 1862.

This is a well written pamphlet, containing many practical suggestions of value to physicians. It commences by giving the practice as adopted by Sydenham, and saying that it is essentially the treatment of the present time; that little if any improvement or change has been made in the general or constitutional treatment of small pox since his time. The treatment of small pox of that time, was, that the initiatory or febrile stage of the disease should be treated with purgatives, cool drinks, neutral mixtures and light farinaceous diet; that at the introduction of the papular stage, the neutral mixture may be withdrawn, the purgatives and farinaceous diet continued; and this treatment must be pursued through the papular, vescular, pustular and desiccating stages, unless the so-called secondary fever should spring up, when the use of the neutral mixtures must be resumed, a tapioca and gruel diet given, and the whole treament, medicinal and dietetic, must be decidedly antiphlogistic, unless, however, typhus symptoms present themselves, when a little beef-tea or small quantity of milk and a little wine-whey and some tonic may be administered. This is the Sydenham treatment—the treatment, as he claims, of the present time, taught by the schools, adopted in small pox hospitals, and set forth in all books which contain articles upon variola. Our author finds no fault with this treatment so far as it relates to the initiatory or febrile stage, beyond that deems the treatment defective, inefficient, pernicious, and finally, does not refuse to say, destructive. He reviews the ground for this treatment, and says it has been developed out of the idea that small pox is an inflammatory affection, and hence demands in all its stages a marked and decidedly antiphlogistic or anti-inflammatory medicinal and dietetic treatment. This view of the nature of small pox he regards as an error, that any inflammatory action which may spring up is accidental, and not an inherent part of small pox; but that it is a *suppurative disease*, and that the arythema or inflammation produced in the tissues involved, is *necessary* and *required* to the safe development and passage of the disease through all its varied and oft-times severe and exhausting stages; that small pox is not an inflammatory disease. This point is dwelt upon at considerable length, and very fully illustrated and demonstrated.

Dr. Neibinger's practice has been in all cases of small pox, during the initiatory or febrile and papular stages of the disease, to prescribe an antiphlogistic medicinal and dietetic treatment; but to abandon this form of treatment as soon as the papules begin to take upon themselves the vesicular form, and then to commence a treatment which in all its essentials shall be supporting. For food recommends a combination of eggs, milk, sugar and ice; for stimulus, Monongahela whiskey. Has frequently had patients take twelve eggs, three quarts new milk and eight ounces of whiskey daily, for several consecutive days, and then some of his patients barely escaped sinking into their graves. In the treatment of these severe cases he also urges the use of meats, poultry, in short, any food abounding largely in the protein elements.

We cannot follow further in detail our author, and will close by saying that his views are correct and well sustained. And we can find no objection to the paper except that he charges physicians generally with following the practice of the authors of books, which we hope and believe is not true, particularly in treating small pox, as well as many other diseases of similar character. Such papers, with experience and time, will correct the books. We think practitioners are vastly in advance of many books in their practice.

---

